# Forces Bless You: Mechanosensitive Piezo Channels in Gastrointestinal Physiology and Pathology

**DOI:** 10.3390/biom14070804

**Published:** 2024-07-07

**Authors:** Jing Guo, Li Li, Feiyi Chen, Minhan Fu, Cheng Cheng, Meizi Wang, Jun Hu, Lixia Pei, Jianhua Sun

**Affiliations:** 1 Health and Rehabilitation College, Nanjing University of Chinese Medicine, Nanjing 210023, China; janiceguo@njucm.edu.cn (J.G.); chengcheng@njucm.edu.cn (C.C.); meiziwang@njucm.edu.cn (M.W.); junhu@njucm.edu.cn (J.H.); 2 Department of Acupuncture and Rehabilitation, The Affiliated Hospital, Nanjing University of Chinese Medicine, Nanjing 210029, China; lilihn@njucm.edu.cn (L.L.); fy_feiyichen@njucm.edu.cn (F.C.); 039318118@njucm.edu.cn (M.F.)

**Keywords:** Piezo channels, mechanosensation, gastrointestinal function, gastrointestinal disorders, calcium influx

## Abstract

The gastrointestinal (GI) tract is an organ actively involved in mechanical processes, where it detects forces via a mechanosensation mechanism. Mechanosensation relies on specialized cells termed mechanoreceptors, which convert mechanical forces into electrochemical signals via mechanosensors. The mechanosensitive Piezo1 and Piezo2 are widely expressed in various mechanosensitive cells that respond to GI mechanical forces by altering transmembrane ionic currents, such as epithelial cells, enterochromaffin cells, and intrinsic and extrinsic enteric neurons. This review highlights recent research advances on mechanosensitive Piezo channels in GI physiology and pathology. Specifically, the latest insights on the role of Piezo channels in the intestinal barrier, GI motility, and intestinal mechanosensation are summarized. Additionally, an overview of Piezo channels in the pathogenesis of GI disorders, including irritable bowel syndrome, inflammatory bowel disease, and GI cancers, is provided. Overall, the presence of mechanosensitive Piezo channels offers a promising new perspective for the treatment of various GI disorders.

## 1. Introduction

The gastrointestinal (GI) tract is a mechanically active organ in which all cells can sense forces emanating from the luminal contents and organ activity. The GI organs conduct a series of precisely calibrated mechanical actions that rely on sensing mechanical forces to provide feedback and ensure effective coordination. The GI tract detects forces through mechanosensation, a mechanism that relies on specialized cells known as mechanoreceptors. These mechanoreceptors utilize mechanosensors to convert mechanical forces into electrochemical signals [1]. The GI tract contains layers of varying physical properties, which allows GI tract mechanoreceptors to effectively sense a broad spectrum of intrinsic and extrinsic mechanical forces [2]. Mechanoreceptors can detect the resultant wall tension originating from tone, displacement, or both, in addition to the shear force resulting from the relative sliding of layers and the luminal flow induced by these actions.

An integral GI tract component, the mechanosensitive cells extend throughout all GI tract layers: the mucosa, submucosa, smooth muscle, and the submucosal and myenteric plexuses [3]. The specialized GI tract mechanoreceptor cells, whose primary function is converting mechanical stimuli into physiological signals, encompass epithelial cells, enterochromaffin (EC) cells, and neuronal cells [2,4,5]. Neuronal cells, including intrinsic primary afferent neurons (IPANs), intestinofugal afferent neurons (IFANs), and extrinsic sensory neurons, are GI tract mechanosensitive cells. The gut contains five structurally distinct types of extrinsic primary afferent endings that determine gut wall mechanics: the intraganglionic laminar endings (IGLEs), mucosal, muscular–mucosal, intramuscular arrays (IMAs), and vascular afferents [6]. Non-specialized mechanosensory cells, such as the interstitial cells of Cajal, smooth muscle cells, glia, and immune cells, use mechanosensors to detect mechanical stimuli and adjust their function accordingly based on the physical condition of their surroundings [2]. Thus, it must be emphasized that responding to mechanical forces is not only crucial but is also a fundamental aspect of normal GI functions.

Sensation and response to mechanical forces are mediated by various proteins, including cytoskeletons, focal adhesion–associated molecules, G protein-coupled receptors, and ion channels [7,8,9,10]. Among these proteins, the mechanosensitive ion channels are crucial, as they can be directly activated by stresses exerted on the lipid bilayer or its associated nonmembrane components [11]. The process of mechanotransduction is rapid, indicating the need for direct gating of transducing channels rather than relying on activation through chemical intermediates [10]. In the GI tract, several mechanosensitive ion channels have been identified involving GI physiology and pathology, including the transient receptor potential (TRP) family, two pore-domain potassium channels (K2p), voltage-gated sodium (Nav) and calcium (Cav) channels, Ca^2+^-activated large-conductance potassium channel (BKCa), Piezo channels, and others.

The recognition of mechanosensing has significantly advanced with the recent identification of Piezo channels, a group of mechano-gated ion channels that are evolutionarily conserved. In 2010, researchers in Ardem Patapoutian’s laboratory discovered the Piezo1 and Piezo2 proteins, encoded by the *PIEZO1/FAM38A* and *PIEZO2/FAM38B* genes, respectively [12]. They utilized RNA interference techniques and whole-cell recording of mechanically activated currents to uncover the crucial role of the *PIEZO1* gene in facilitating the intrinsic mechanically activated cationic currents in the mouse Neuro2A neuroblastoma cell line. Furthermore, upon examination of sequence homology, the existence of the homologous gene *PIEZO2* was revealed [12]. Subsequently, a large number of related studies have been published that extensively explored the structure, mechanogating mechanism, distribution, and physiopathology associated with these molecules (Table 1).

Piezo channels are expressed in numerous cells of the GI tract and contribute significantly to their proper functioning. Several studies have provided increasing evidence to highlight the crucial role of Piezo channels in the GI tract [3,13,14,15]. Here, the current review focuses on current studies that examine the influence of Piezo channels on GI functions. Specifically, the crucial role these channels play in the intestinal barrier, GI motility, and intestinal mechanosensation regulation is emphasized. Furthermore, the current understanding of mechanically activated Piezo channels in the pathogenesis of GI disorders, including irritable bowel syndrome (IBS), inflammatory bowel disease (IBD), and GI cancers, is examined in detail.

**Table 1:** Similarities and differences between Piezo1 and Piezo2.

Items	Piezo1	Piezo2	Reference
Gene	Fam38A	Fam38B	[12]
Chromosomal localization	human chromosome 16	human chromosome 18	NCBI database (https://www.ncbi.nlm.nih.gov/gene/9780 accessed on 17 June 2024. https://www.ncbi.nlm.nih.gov/gene/63895 accessed on 17 June 2024.)
Gene region	16q24.3	18p11.22-p11.21	
Amino acid size in human	2521 amino acids	2752 amino acids	[16]
Amino acid size in mice	2547 amino acids	2822 amino acids	[17,18]
Tissue distribution	skin, bladder, kidney, lung, endothelial cells, erythrocytes, periodontal ligament cells, etc.	trigeminal sensory neurons, dorsal root ganglion, Merkel cells, somatic neuron cells, etc.	[19]
Delection threshold (fJ)	213.7 ± 16.6	86.8 ± 7.1	[20]
Work resolution (fJ)	1.2 + 0.4	1.0 + 0.2	[20]
Transduction speed (ms)	8.2 ± 2.2	1.5 ± 0.5	[20]
Inactivation kinetics (ms)	16.5 ± 1.4	7.3 ± 0.7	[12]
Structure	a homotrimer structure resembling a three—bladed propeller		[18,21]
Function	involving in mechanotransduction in various non-excitable cell types	sensing slight touch and proprioception	[22]
Activator	Yoda1, Jedi1/2	not found yet	[23,24]
Inhibitor	Ruthenium red (RR), Gadolinium (Gd^3+^), Dooku1, and GsMTx4	RR, Gd^3+^, and GsMTx4	[25,26,27,28,29]
Hereditary human disorders	dehydrated hereditary stomatocytosis, generalized lymphatic dysplasia, etc.	distal arthrogryposis, Gordon syndrome, Marden-Walker Syndrome, etc.	[30,31,32,33]

## 2. Piezo Channels in Mechanosensory Cells That Detect Gastrointestinal Forces

### 2.1. Epithelial Touch Sensors

**Figure 1:**
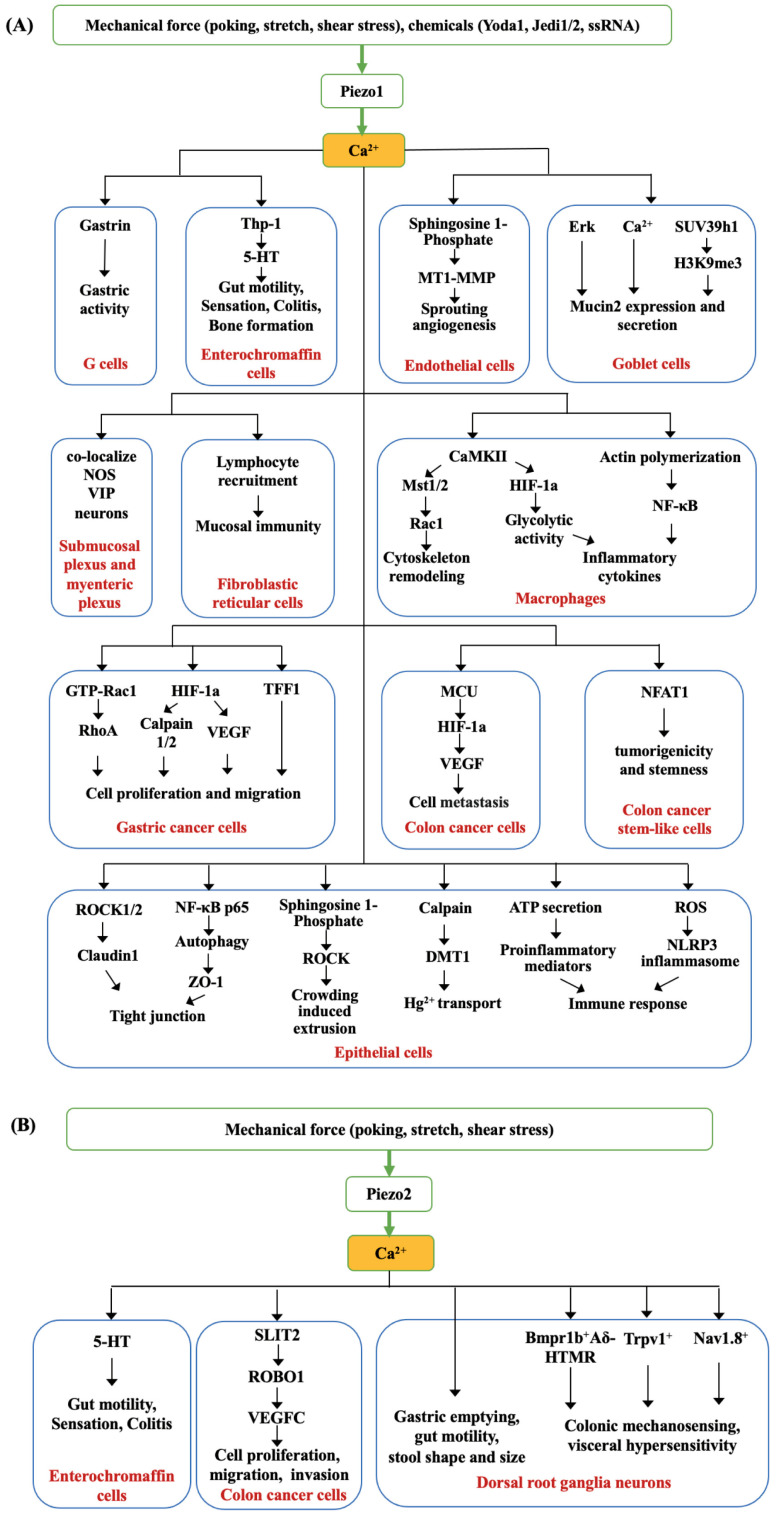
Piezo1- and Piezo2-mediated mechanotransduction in gastrointestinal tract. (A) Piezo1 functions as a mechanotransduction channel for mediating distinct Ca^2+^ signaling pathways in various gastrointestinal cell types. (B) Piezo2 is a mechanotransduction channel for initiating Ca^2+^ in primary sensory neurons and specialized cell types, such as enterochromaffin cells and colon cancer cells.

**Figure 2:**
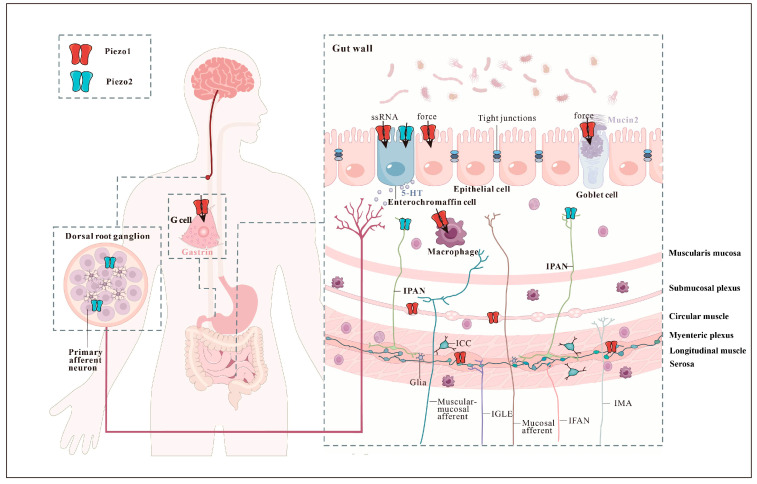
Schematic depiction of the distribution of mechanosensitive Piezo1 and Piezo2 in the gastrointestinal tract.

Piezo1 and Piezo2 are widely expressed in various parts of the GI tract. It is important to examine their expression in different regions to gain a more detailed understanding of their specific localization (Figure 1 and Figure 2). The stomach contains a notable abundance of Piezo1 in the basolateral aspect of G cells, which produce gastrin and control gastric activities [34]. Further down the GI tract, the intestinal epithelial cells (IECs) act as the initial barrier in responding to the gut lumen pressure and contents [35]. Interestingly, Piezo1 is highly expressed in several subtypes of IECs, such as epithelial cells, goblet cells, and enteroendocrine cells [36,37,38].

An enteroendocrine cell subtype, known as EC cells, is suggested to function as specialized epithelial mechanosensors. These cells can produce a large outflow of serotonin (5-HT) in response to mucosal forces. It is noteworthy that EC cells have developmental and functional similarities with Merkel cells, the touch sensors of the skin. Piezo2 channels serve as the primary mechanosensors in both Merkel and EC cells. EC cells express Piezo2 [27,39,40,41], which is close to cortical filamentous actin (F-actin) and in close apposition to 5-HT-containing vesicles, suggesting membrane localization and functional coupling, respectively [40]. Upon activation by mechanical forces, Piezo2 channels in EC cells produce an ionic current that leads to an increase in intracellular Ca^2+^, 5-HT release, and epithelial fluid secretion [40].

Another interesting study unexpectedly suggested that Piezo1 channels in EC cells function as fecal single-stranded RNA (ssRNA) sensors rather than force sensors. According to the study, the fecal ssRNA–Piezo1 axis is pivotal in the microbiome response and gut motility, mediated by systemic 5-HT synthesis [36]. Nevertheless, the specific location of the Piezo1 channel in the epithelial layer or the EC cells is unclear. Further experiments employing more advanced tools or direct recordings from in situ cells are required to investigate where Piezo1 functions primarily [42]. Moreover, it would be of interest to determine whether Piezo1 responds to ssRNA species from non-bacterial organisms [43].

### 2.2. Mechanosensitive Enteric Neurons

The search for mechanoreceptors and mechanotransducers in enteric nerves is continuously evolving. The potential candidates responsible for mechanosensitive enteric neuron (MEN) mechanotransduction include the Piezo channels. Specifically, Piezo1 is expressed in the soma and neurites of the myenteric and submucosal plexi. Myenteric Piezo1 is predominantly expressed in enteric nitrergic and some cholinergic enteric neurons. Likewise, in the submucosal plexus, Piezo1 neurons co-express vasoactive intestinal peptide [44].

Conversely, Piezo2 expression is scarce in enteric neuronal soma but can be detected in a few neurites [44,45]. The mechanosensitive and Piezo1-positive neurons were significantly positively correlated [44]. However, pharmacological experiments using the Piezo1 inhibitor GsMTx4 did not affect mechanotransduction, suggesting that Piezo1 may not play a major role in the mechanosensitivity of enteric neurons [44]. A detailed examination of the human and mouse enteric nervous system (ENS) at the single-cell level indicated that putative motor neurons express Piezo1, while putative sensory neurons express Piezo2. The specific role of Piezo channels in regulating MEN mechanotransduction warrants further investigation.

### 2.3. Intrinsic Primary Afferent Neurons

IPANs correspond to cells with Dogiel type II morphology and feature large and smooth cell bodies in the submucosal and myenteric plexuses along the entire length of the GI tract [46]. A study using single-cell RNA sequencing defined a molecular taxonomy of 12 enteric neuron classes (ENC) within the myenteric plexus of the mouse small intestine. The particular morphology displayed by a subset of ENC12 neurons and the expression of the mechanosensory channel Piezo2 connected potential mechanosensory to the reported filamentous IPANs [47]. Further work is required to ascertain the identity of Piezo2 in the IPAN mechanosensing.

### 2.4. Extrinsic Sensory Neurons

From the central nervous system (CNS) perspective, GI sensory information is transmitted to the brainstem and spinal cord via the vagal afferent nerve and the spinal afferent nerve (splanchnic and pelvic) [6]. Extrinsic vagal afferent nerve endings largely innervate the upper gut (esophagus and stomach), and spinal afferent nerve endings, whose cell bodies lie in the dorsal root ganglia (DRG), provide extremely rich sensory innervation to the lower gut (colon) [48]. The colon-innervating neurons include a population of the DRG sensory neurons that mediate pain, DRG sensory neurons and enteric neurons, and sympathetic neurons [49].

Piezo2 is highly expressed in the DRGs. It serves as a major mechanically sensitive cation channel in low-threshold mechanoreceptors (LTMRs) and is essential for touch sensation [50,51]. Additionally, Piezo2 is also expressed by parvalbumin-expressing neurons involved in proprioception [52], as well as in nociceptors, marked by TRPV1 or VGLU3, which are involved in mechanical pain [12,50,53]. Recent studies have highlighted the significance of Piezo2 in DRG mechanotransduction, influencing the conversion of somatosensory information into visceral sensation [45,49,54,55]. Transcriptomic profiling of colon-projecting DRG neurons in mice identified seven colonic sensory neuron classes, with both the mNonPeptidergic and mNeuroFilament subtypes expressing Piezo2 [56]. However, the specific functions of Piezo2 in each subtype remain to be tested. Piezo2-expressing DRG neurons directly project into the GI tract with a predominant morphology of intraganglionic varicose endings (IGVEs), and the colon presents the highest innervation density [49,57]. A single-cell qRT-PCR analysis revealed that Piezo2 mRNA transcripts were detected in 53.6% of the colon-innervating lumbosacral DRG neurons [58]. Moreover, Piezo2 is functionally expressed in TRPV1-lineage neurons [58], Nav1.8-expressing nociceptors [45], and Bmpr1b^+^ Aδ-high-threshold mechanoreceptors (HTMRs) [59], indicating the critical role of this channel in mechanosensitivity and nociception.

## 3. Mechanosensitive Piezo Channels Affect the Gastrointestinal Function

### 3.1. Intestinal Barrier

The mechanical barrier is mainly comprised of a single layer of IECs between the mucus barrier and the immune barrier, which is an impenetrable physical barrier that can limit the interaction between the luminal contents and the body environment [60]. Previous studies suggested that Piezo1 in the epithelial cells might be closely associated with tight junctions. Piezo1 partially regulates intestinal epithelial dysfunction via claudin1 through the ROCK1/2 pathway. Knocking down or inhibiting Piezo1 enhanced epithelial barrier integrity, while Piezo1 overexpression or activation induced the side effects [61]. Piezo1 also activates IEC autophagy and promotes zonula occludin (ZO)-1 expression to maintain intestinal barrier function, which is regulated by the NF-κB signaling pathway [62]. Furthermore, the quantity and structure of epithelial cells are also critical for mechanical barrier function, which can be maintained by Piezo1-induced extrusion of living cells [63]. Overcrowding caused by proliferation and migration can induce the extrusion of live cells from the epithelial cells of human colonic tissues. The process is activated by the stretch-activated channel Piezo1, through which sphingosine 1-phosphate signaling and ROCK-dependent myosin contraction occur to control epithelial cell numbers [64]. Additionally, Piezo1 is actively involved in the adaptive mechanism of live cell extrusion when overcrowding occurs in tissues, which maintains epithelial integrity during development and homeostasis [65].

The chemical barrier (mucus barrier) is a viscous gel-type network structure mainly composed of mucins, water, antimicrobial peptides, and secretory immunoglobulin A [66,67]. Goblet cells are the major contributors to mucus layer secretion, which includes densely glycosylated mucin2 [68]. Secreted mucin2 is stabilized by intramolecular cross-linking to form gel mucus [69,70]. Piezo1 is abundantly expressed in goblet cells. Mechanical stimulation models in vivo and in vitro demonstrated that the Piezo1 protein in goblet cells sensed mechanical stimulation and regulated mucin2 synthesis and secretion via the downstream Erk/Ca^2+^ pathway [37]. Moreover, the epigenetic mechanisms of Piezo1’s functions in goblet cells have been investigated. Specifically, in water avoidance stress (WAS) mice, a decline in goblet cell populations and Piezo1 levels was observed. Piezo1 is crucial in transmitting mechanical stimulation to inhibit histone deacetylase 3 (Hdac3) and methyltransferase suppressor of variegation 3–9 homolog 1 (SUV39h1), thereby decreasing the production of histone H3 lysine 9 trimethylation (H3K9me3). Furthermore, Piezo1 hinders H3K9me3 binding to the mucin2 promoter, effectively augmenting mucin2 expression in goblet cells [38].

In goblet cells, Piezo1 plays a pivotal role not only in sustaining the normal function of the mucus layer but also in preserving the microbial flora structure, which is intricately linked to the mucus layer’s function. *Piezo1*^–/–^ mice exhibited elevated proinflammatory cytokine expression, coupled with a notable decrease in mucin2 and AGR2 expression. This led to a reduction in mucus thickness and goblet cell numbers in the colon, ultimately triggering long-term spontaneous inflammation [71]. Specifically, alpha-diversity analysis revealed a downregulation of the fecal-associated microbiota composition of *Piezo1^–/–^* mice, while the mucosa-associated microbiota underwent an upregulation [71]. Therefore, Piezo1 in goblet cells can influence the microbial community structure and regulate gut microbiota homeostasis.

The immune barrier beneath the physical barrier recognizes pathogens and foreign bodies, producing antibodies and cytokines and regulating the immune response. IECs sense infections via Piezo1, which detects infection-induced ruffles and mediates Ca^2+^ influx and ATP release, ultimately imitating acute inflammation [72]. Furthermore, blood vessels and lymphatic vessels are channels that transport immune cells and other immune-related substances. Both mechanical and biochemical stimuli trigger Piezo1-mediated Ca^2+^ influx, leading to the activation of matrix metalloproteinase-2 and membrane type 1 matrix metalloproteinase. This activation acts synergistically to promote sprouting angiogenesis [73]. The fibroblastic reticular cell (FRC) conduit networks in the intestinal Peyer’s patches (PPs) maintain the normal structure of high endothelial venules and lymphocyte recruitment by conducting the direct flow of absorbed lumenal fluid via Piezo1 [74].

Pathogens stimulate toll-like receptor 4 (TLR4) to induce Piezo1-mediated Ca^2+^ influx and remodel F-actin by activating the Ca^2+^/calmodulin-dependent kinase II (CaMKII)–Mst1/2–Rac1 axis. Consequently, this pathway augments the bactericidal effect of macrophages for pathogen ingestion and killing [75]. Another study reported that Piezo1 is involved in regulating Ca^2+^ influx-induced CaMKII activation to promote the release of hypoxia-inducing factor 1a (HIF-1a) to regulate macrophage glycolytic activity, thereby enhancing macrophage inflammation [76]. Notably, Piezo1 serves as a mechanical stiffness sensor in macrophages, regulating the macrophage polarization response and participating in the perception of microenvironmental stiffness. Piezo1 activation by IFNγ/lipopolysaccharide (LPS) on stiff substrates promotes actin polymerization, thus enhancing Ca^2+^ influx. This positive regulation between actin and Piezo1 enhances inflammation via NF-κB activation and the subsequent upregulation of inflammatory cytokines [77].

### 3.2. Gastrointestinal Motility

Mechanosensitive Piezo channels primarily affect GI motility by releasing 5-HT from EC cells and acting on the DRG to regulate GI function. Piezo2 influences 5-HT secretion by EC cells through its classical mechanical sensing function, which includes promoting intestinal motility [78]. A recent study demonstrated slower whole gut transit and colon transit times in an epithelial-specific *Piezo2* knockout model as compared to wild-type animals [79]. CNO-mediated activation of Piezo2 cells caused epithelial EC cells to release large amounts of 5-HT [39]. Mice treated with CNO-activated Piezo2 cells exhibited a notable increase in fecal particle output within 15 min post-administration. Additionally, these mice demonstrated a significantly shorter time for discharging the inserted bead during the bead expulsion assay, indicating remarkably accelerated colon movement [39]. Intriguingly, 5-HT induction after cyclic stretch was Piezo2-dependent but Piezo1-independent, suggesting that Piezo1 acts unconventionally [40]. Piezo1 senses luminal microbial ssRNA and regulates the transcription of tryptophan hydroxylase 1 (Thp1), a key enzyme for producing 5-HT, which is released to enhance intestinal motility [43].

In the DRG, Piezo2 is necessary for the perception of the intestinal contents and the slowing of food transit speeds in the stomach, intestine, and colon. Servin-Vences et al. assessed the GI health and medical history of seven human subjects carrying *Piezo2* loss-of-function variants, in contrast to 1,177 healthy control participants. Their study revealed that *Piezo2*-deficient individuals exhibited GI abnormalities across diverse age demographics, indicating impaired sensation in bowel movements [57]. Furthermore, these individuals reported an elevated incidence of both watery stools and constipation, suggesting that the Piezo2 channel might have a multifaceted role in regulating GI motility.

Piezo2 sensory endings of DRG origin innervate the stomach, intestine, and colon with a predominant IGVE morphology. Servin-Vences et al. performed ablation of the *Piezo2* gene from several cell classes that can sense mechanical signals from the gut, including the vagal afferents (nodose ganglia), spinal afferents (DRG), gut epithelial cells, and ENS [57]. Subsequently, they measured the influence of the ablation on the time taken for ingested food to be excreted. Mice with *Piezo2*-deficient DRG neurons had significantly reduced GI transport times and increased defecation frequency as compared with the wild-type littermate controls. Notably, only the ablation of *Piezo2*, specifically in DRG neurons, affected GI transit as it accelerated. Interestingly, a Piezo2-dependent slowdown in gut transit occurred exclusively in fed animals but not in fasted ones. *Piezo2*-knockout mice exhibited delayed evacuation and expelled larger beads (3 and 4 mm) in bead expulsion assays, suggesting an additional role of Piezo2 in regulating stool shape and size. When Piezo2 was removed from the DRG, all responses to colonic stimulation (brush insertion and withdrawal and balloon inflation) were eliminated, with only the response to painful squeezing remaining, which suggests that Piezo2 in DRG neurons is a key colonic stretch sensor. The results suggested that the absence of Piezo2 interferes with stretching detection, thereby delaying the onset of mechanical peristalsis in the rectum. Taken together, Piezo2 in DRG neurons is essential for sensing gut contents and decreasing food transit rates in the stomach, small intestine, and colon.

### 3.3. Intestinal Mechanosensation

Mechanosensitive Piezo channels mediate visceral sensation directly via spinal sensory neurons or indirectly via 5-HT released by EC cells. Several studies have demonstrated a prime role for Piezo2 in colon-innervating DRG neurons during gut mechanosensation. DRG neurons detect colon distension via Piezo2. Reducing Piezo2 levels in DRG via intrathecal Piezo2-short hairpin RNA (shRNA) attenuated visceral sensation to innocuous stimuli in wild-type rats. The relief of visceral sensation to both innocuous and noxious stimuli was observed when using intrathecal Piezo2-shRNA in visceral hypersensitivity rats [54]. The Piezo2-knockout animals had significantly diminished DRG neuron responses to colon distension. The phenotypes from the conditional deletion of *Piezo2* in neurons expressing Scn10a or TRPV1 were similar to those observed when knocking out this mechanoreceptor in all DRG neurons [57,58]. This result suggested that Piezo2 might play a potential role in gut mechano-nociception. However, Piezo2 expressed in the Bmpr1b^+^Aδ-HTMR population contributed to the high-threshold colon distension but was not fully responsible for it. Deleting *Piezo2* did not eliminate the behavioral responses to high-threshold distension, suggesting that another sensor might detect either high-threshold stretch or tissue damage [59,80].

Intriguingly, a recent study reported a novel phenomenon, indicating that Piezo2 functions differently in males and females in terms of colonic mechanosensing and pain hypersensitivity [45]. Female mice exhibited significantly more Piezo2-expressing DRG neurons, wherein the soma size was notably larger compared to that of male mice. Furthermore, the proportion of Piezo2-expressing Nav1.8-lineage DRG neurons was higher in female mice compared to male mice. The chemogenetic activation findings from Piezo2-expressing cells, or DRG neurons, aligned with the results obtained from localized optogenetic activation of Piezo2-expressing thoracolumbar DRG neurons. This result suggested that activating Piezo2-expressing sensory neurons induces colonic hypersensitivity in male but not female mice. Conditional deletion of *Piezo2* from Nav1.8 nociceptive neurons results in reduced colonic mechanical sensitivity, specifically in female mice. However, it should be noted that Piezo2 in nociceptive neurons is involved in the development of colonic hypersensitivity and pain-sensing in male mice and not in female mice. These results suggested that Piezo2 in nociceptive neurons mediates innocuous colonic mechanosensing in female mice and painful sensation in male mice. Future studies are needed to unravel the intricate molecular mechanisms of Piezo2 that drive sex differences in mechanosensory responses.

5-HT released from EC cells regulates GI activity via 5-HT receptor subtypes expressed on intrinsic and extrinsic primary afferent neurons [81]. In mouse intestinal tissue sections, 5-HT_3_ receptor-expressing nerve fibers were detected immediately adjacent to EC cells and formed synaptic-like structures for transmitting signals in a restricted, point-to-point manner. EC cells form synaptic contact with sensory neurons, utilizing 5-HT as a neurotransmitter [82,83]. Piezo2 channel activation by mechanical forces or Piezo1 in response to fecal ssRNA modulates Ca^2+^ influx to induce 5-HT release [36,40]. 5-HT modulates the function of nearby neurons and sensory information transmission from the viscera to the brain and spinal cord via vagal and spinal afferents.

## 4. Mechanosensitive Piezo Channels in the Gastrointestinal Disorders

### 4.1. Irritable Bowel Syndrome

IBS is a widespread functional GI disorder, characterized by abdominal pain, bloating, bowel movement frequency, and abnormal stool form [84]. The prevalence of IBS varies significantly depending on the diagnostic criteria used. IBS is estimated to affect 3.8–9.2% of the general population [85]. IBS significantly impacts individuals’ quality of life, healthcare delivery, and society as a whole in terms of economic costs [86]. Abdominal pain is considered the core symptom of IBS, and its severity is the main driving factor in patients’ medical treatment behavior [87].

**Figure 3:**
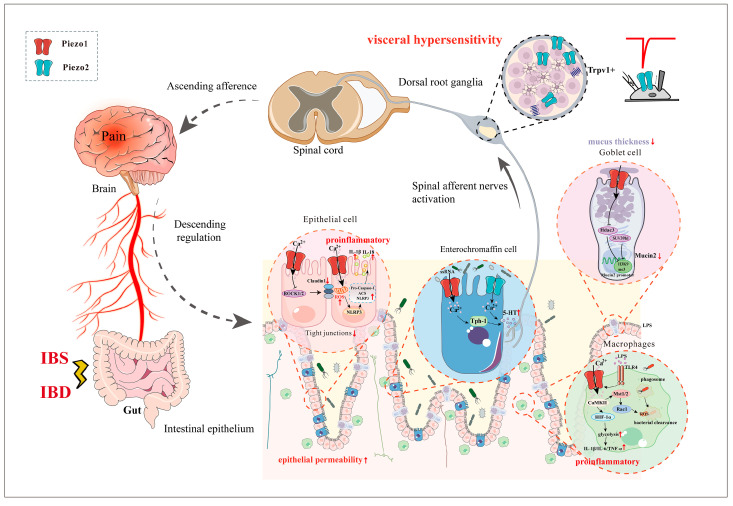
Piezo1 and Piezo2 in irritable bowel syndrome (IBS) and inflammatory bowel disease (IBD).

IBS is commonly associated with an impaired intestinal barrier. Piezo1 expression is significantly elevated in the intestinal epithelium, directly affecting intestinal barrier function and permeability. Specifically, Piezo1 partially controls ROCK1/2, which in turn modulates claudin1 expression and ultimately negatively affects epithelial barrier function [61]. Additionally, activation of Piezo1 in goblet cells reduced methyltransferase SUV39h1 expression, which decreased methylated histone H3K9me3 production and its binding to the mucin2 promoter, a core component of the mucus barrier [38]. This change ultimately promoted mucin2 production and significantly affected mucus barrier function (Figure 3).

Piezo2 expression in the colon epithelial cells was significantly correlated with visceral sensitivity in post-infectious IBS (PI-IBS) model mice induced via a *Trichinella spiralis* infection, which indicated that Piezo2 is a potential biomarker for visceral hypersensitivity in IBS [88]. Moreover, individuals with IBS exhibit heightened production of peripheral 5-HT originating from intestinal EC cells, which is significantly linked to the severity of their symptoms [89,90]. Therapeutic interventions that include 5-HT_3_ receptor antagonists and 5-HT_4_ receptor agonists have demonstrated promising clinical efficacy in managing either diarrhea or constipation-predominant IBS, respectively [91]. EC cells detect environmental and endogenous stimuli capable of eliciting or exacerbating pain, including microbial metabolites via the Piezo1 channel and mechanical distension via the Piezo2 channel [36,40]. Prolonged EC cell activation led to the development of persistent visceral hypersensitivity [92]. Once activated through mechanosensitive Piezo channels, the EC cells release 5-HT onto nearby 5-HT_3_ receptor-expressing sensory nerve endings, which then transmit nociceptive signals to the spinal cord [83,93]. Thus, the EC cell–mucosal afferent nerve circuit is critical in acute and persistent GI pain [92,94] (Figure 3).

Sensory nerve endings can directly detect noxious stimuli [95]. Distinct types of spinal afferents innervate the gut and respond to varying distension thresholds [59]. High Piezo2 expression in the DRG and its colocalization with small-diameter unmyelinated nociceptors suggest a possible role in the distal colon response to noxious mechanical stimuli [19]. It is notable that colonic balloon distention produces forces that fall within the noxious range, causing heightened nociception in IBS. Piezo2 activation in sensory neurons evokes colonic hypersensitivity, while *Piezo2* deletion or knockdown protects against pain sensitization [45,54,58]. In a rat model, intracolonic infusion of acetic acid-induced visceral hypersensitivity was attenuated by *Piezo2* knockdown in the DRG, indicating the potential role of Piezo2 in mediating the hypersensitivity response [54]. PI-IBS induced by 2,4,6-trinitrobenzene sulfonic acid (TNBS) led to colonic mechanical hypersensitivity and upregulated Piezo2 expression in the DRG [96].

Xie et al. provided compelling evidence that the mechanosensitive Piezo2 channels expressed by TRPV1-lineage nociceptors are involved in the perception of mechanical pain in the viscera under pathological conditions [58]. Piezo2 in the DRG neurons was significantly increased in IBS model mice, induced by zymosan, an inflammatory yeast cell wall derivative commonly used to induce visceral hypersensitivity. When the zymosan model was established in Trpv1^Cre^::Piezo2^fl/fl^ mice, the *Piezo2* conditional knockout mice had both markedly lower ex vivo circumferential stretch-evoked firing- and in vivo CRD (colorectal distension)-induced visceromotor responses [58]. Deleting *PIEZO2* decreased Ca^2+^ channel activity and reduced CREB and Akt activity in DRG neurons. Additionally, *Piezo2* deletion decreased CGRP central release, ultimately leading to spinal central sensitization in response to noxious CRD-induced colonic pain [45]. The mechanosensitive Piezo2 channels involved in visceral mechanical nociception and hypersensitivity might be a new therapeutic target for IBS-related gut pain [55] (Figure 3).

### 4.2. Inflammatory Bowel Disease

IBD is a group of immune system-mediated inflammatory diseases that affect the GI tract. Recently, chronic idiopathic disorders have gained prominence due to their ability to cause GI tract inflammation [97]. This condition has become a significant public health concern globally, with its prevalence increasing over the past decade [98]. The main manifestations of IBD are Crohn’s disease (CD) and ulcerative colitis (UC).

CD is an incurable chronic intestinal disease with symptoms that include diarrhea, abdominal pain, and weight loss [99]. Chronic intestinal inflammation is a key mechanism in CD. The ileum of active CD patients had significantly increased Piezo1 protein expression levels, suggesting its close association with intestinal inflammation. In vitro experiments were conducted to investigate the role of Piezo1 in epithelial cells, specifically its involvement in inflammatory cytokine release [100]. The results demonstrated that increased Piezo1 expression led to Ca^2+^ influx in HT29 cells, resulting in mitochondrial dysfunction characterized by reactive oxygen species (ROS) accumulation and decreased membrane potential, thus activating the NLRP3 inflammasome and exacerbating intestinal inflammation [100]. Overall, these results shed light on the crucial role of Piezo1 in CD pathogenesis (Figure 3). Consequently, they provided critical insights for further research on the underlying mechanisms of CD and the development of novel therapeutic strategies. What’s more, CD4+T cell activation is a key feature of IBD. In T cell-specific *Piezo1* knockout mice, the loss of Piezo1 promoted Th1 and Th17 cell polarization, while the subsequent reduction in inflammatory signaling suggested that Piezo1 has an important regulatory role in the inflammatory response of pathogenic T cells [101]. Thus, Piezo1 plays a crucial role in intestinal inflammation.

Dextran sulfate sodium (DSS)-induced UC in mice is a self-resolving condition. Piezo1 exists at the infectious site in UC. Piezo1 modulates aerobic glycolysis in macrophages and enhances the LPS-induced immune response. Furthermore, Piezo1 can increase IL-6, TNF-α, and IL-1β levels [76]. Therefore, Piezo1 is important in intestinal inflammation through immune factors. Moreover, Piezo1 activation in IECs is also important for the reprogramming of inflammatory and immune genes. Piezo1 induces Ca^2+^ influx and leads to ATP secretion, ultimately affecting inflammatory factor expression in IECs. Additionally, activated Piezo1 promotes the secretion of immune factors such as IL-8 and controls regulatory genes in immune pathways [72]. Ultimately, Piezo1 affects inflammation and immune responses in the gut.

### 4.3. Gastrointestinal Cancers

**Figure 4:**
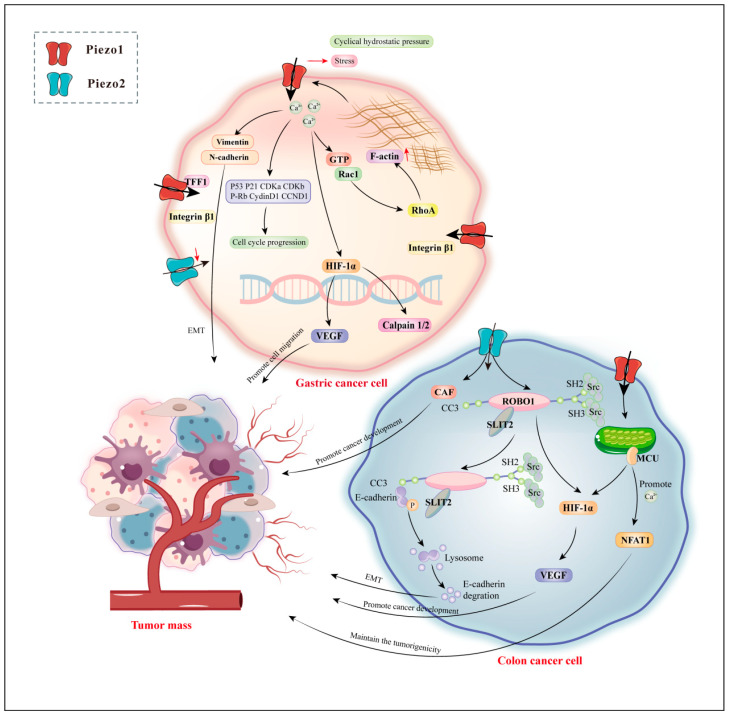
Piezo1 and Piezo2 in gastric cancer and colon cancer.

Carcinogenesis is characterized by changes in the mechanical properties of the affected tissue. Mechanotransduction disturbances have been identified as potential contributors to the development of gastric cancer (GC) and colon cancer. Several studies have demonstrated that Piezo channel activation modulates the Ca^2+^-dependent signaling pathways linked to cancer cell migration, proliferation, and angiogenesis [102]. Disturbed mechanotransduction in the GI tract has been implicated in GC and colon cancer. GC is the fifth most frequently diagnosed malignancy globally and is the third leading cause of cancer-related mortality worldwide [103,104]. Piezo channels have increased potential for GC-targeted markers and therapy (Figure 4). Clinically, Piezo2 expression is lower in GC tissues than in adjacent normal tissues and is correlated with lymph node metastasis and tumor node metastasis (TNM) classification stages in patients with GC. Piezo2 is a potentially poor prognostic and diagnostic biomarker or therapeutic target in patients with GC [105].

In contrast to Piezo2, Piezo1 is upregulated in GC cell lines compared with non-tumorous gastric epithelial tissues [106]. Similarly, the Piezo1 expression level in GC tissues with omentum metastasis and metastatic lymph node tissues is higher than that in GC tissues [107]. Moreover, lymph metastasis, TNM, and distant metastasis are associated with high Piezo1 expression. In vitro, Piezo1 overexpression facilitated cell proliferation and suppressed apoptosis. In vivo, Piezo1 knockdown by small interfering RNAs (siRNAs) significantly inhibited or blocked the peritoneal metastasis, epithelial–mesenchymal transition (EMT), and angiogenesis of GC cells [107]. The above studies suggest that Piezo1 is involved in the process of GC-omental transfer. Further studies demonstrated that the transfer process involved hypoxia-inducible factor 1α (HIF-1α) upregulation.

Furthermore, *Piezo1* knockdown cells had increased GTP-Rac1 levels, and GTP-Rac1 overactivation interrupted the GTP-GDP transition cycle and restricted the extent of Rho protein activation. RhoA enhances stress fiber formation, and Rac1 facilitates lamellipodia. The two proteins control cell migration by regulating F-actin movement, polymerization, and depolymerization. Thus, knocking down *Piezo1* leads to dramatic cell shape alteration and limited cell motility [106]. Moreover, the C-terminal portion of the Piezo1 protein interacts with trefoil factor family 1 (TFF1), a member of the TFF-domain peptide family involved in epithelial restitution and cell motility. Thus, Piezo1 is important in TFF1-mediated cell migration and might be a therapeutic target for GC invasion and metastasis. Surprisingly, decreasing Piezo1 enhanced the sensitivity of cisplatin or 5-fluorouracil treatment in vitro [108].

Colon cancer is a tumor that poses a significant threat to human health and detrimentally affects the quality of life. The common symptoms associated with colon cancer include bowel habit changes, abdominal discomfort, weight loss, anemia, and more [109,110]. Notably, high Piezo1 expression is strongly correlated with poorer prognosis in patients with colon cancer (Figure 4). An interesting observation was that Piezo1 overexpression enhanced colon cancer cell survival, migration, and metastasis capabilities. In these cells, Piezo1 activation downregulated the mitochondrial calcium uniporter (MCU) while upregulating HIF-1α [111]. In the tumor environment, hypoxia-induced activity coordinates angiogenesis, EMT, stem cell preservation, invasion, metastasis, and resistance to radiotherapy and chemotherapy [112]. Specifically, VEGF influences tumor angiogenesis and metastasis. It is important to note that Piezo1 expression level is significantly correlated with VEGF expression, vascular invasion, and HIF-1α expression [112]. Additionally, silencing *Piezo1* inhibited HIF-1α and VEGF expression. Thus, Piezo1 promotes colon cancer development by regulating the Piezo1–MCU–HIF-1α–VEGF signaling pathway [111]. Another study demonstrated a positive correlation between Piezo2 and the expression of VEGFC and HIF-1α. This association suggested that Piezo2 is potentially involved in the SLIT2–ROBO1–VEGFC signaling pathway. SLIT2–ROBO1 signaling pathway activation stimulates the transforming growth factor β (TGF-β)–Smads pathway [113]. Additionally, pathway activation induces E-cadherin degradation and promotes EMT by recruiting Src to E-cadherin. Furthermore, the SLIT2–ROBO1 pathway facilitates blood vessel formation, fulfilling the demands of continuous tumor proliferation and expansion [113]. Moreover, cancer-associated fibroblasts (CAF) are crucial in regulating cancer development within the tumor microenvironment [114,115]. Interestingly, Piezo2 expression is positively correlated with CAF expression in colon cancer [113]. Overall, Piezo1/2 has significant implications for colon cancer treatment, specifically the precise targeting of HIF and VEGF.

Cancer stem cells are crucial in tumor development and treatment, serving as the core cells in these processes [116]. Colon cancer stem cell-like cells (CCSCs) are specifically significant in colon cancer occurrence and progression and have become a major research focus [117]. Piezo1 expression has been closely linked to CCSCs and the staging of colon cancer. Yoda1 induces a rapid increase in intracellular Ca^2+^ levels, leading to nuclear factor of activated T cell 1 (NFAT1) signaling pathway activation [118]. This activation aids in the maintenance of CCSC stem cell properties. Conversely, deleting Piezo1 promotes NFAT1 protein degradation through the proteasome pathway, reducing CCSC tumorigenicity and self-renewal ability [118]. Therefore, Piezo1 is critical in sustaining tumorigenesis and immune resistance within CCSCs, making it a potential therapeutic target for managing CCSCs.

## 5. Conclusions and Perspectives

The recent number of Piezo channel-related publications has demonstrated a strongly increasing trend [119]. Developments in Piezo channel research have deepened our understanding of GI tract mechanobiology. Piezo1 and Piezo2, acting as mechanosensitive transducers physiologically and pathologically, are widely expressed in various GI mechanosensitive cells, including IECs, EC cells, and intrinsic and extrinsic enteric neurons. In the gut, several cells express both Piezo1 and Piezo2, but whether they work together remains unclear. Furthermore, the respective contributions of each isoform to mechanotransduction have not been tested.

Piezo1 is important for intestinal barrier functions. In goblet cells, Piezo1 promotes mucin2 release, which in turn aids in the maintenance of the mucus barrier and balances the intestinal microflora. However, Piezo1 also affects tight junctions negatively, compromising mechanical barrier integrity. In EC cells, Piezo1 evoked by fecal ssRNA and Piezo2 activated by mechanical forces enhance intestinal 5-HT synthesis, regulating gut motility and sensation. The Piezo2 channels expressed in gut-innervating DRG neurons mediate visceral mechanical sensitivity and the sensation of colonic distension. Furthermore, Piezo2 in DRG neurons is essential for slowing down gastric emptying and intestinal transit while also regulating stool shape and size. Further research is necessary to ascertain the precise function of Piezo1/Piezo2 in the defecation process in the human rectum and anus.

Piezo channels are involved in the pathogenesis of GI disorders, including IBS, IBD, GC, and colon cancer. These channels are considered a novel and promising therapeutic strategy for treating GI disorders. However, the transition from animal models to human clinical research in GI applications has been significantly constrained, particularly with regard to *Piezo* gene inheritance patterns and mutations in humans, which are remarkably scarce. This highlights the urgent need for more comprehensive clinical studies on human *Piezo* gene variations to address the current knowledge gap. Moreover, drug design targeting Piezo channels is subject to many challenges and limitations. Currently, studying Piezo2 transduction involves using physical stimulation to deflect cell bodies or neurite membranes [120,121], which greatly limits the types of experiments that can be used to study Piezo2 physiology [122]. Recent research revealed the homotrimeric structure of the mouse Piezo2, resolving it to a range of 3.6–3.8A° [18]. This structural insight could prove valuable for identifying specific compounds targeting the Piezo2 channel. Although these compounds were effective in certain models, it remains unclear whether inhibiting Piezo2 will be well-tolerated. Many antagonists (RR, Gd^3+^, Dooku1, and GsMTx4) and agonists (Yoda1 and Jedi1/2) have recently been developed to directly or indirectly bind Piezo1. However, these modulators generally demonstrate modest binding affinities and non-optimal drug-like properties. Although drugs specifically targeting Piezo channels must undergo a long journey before they reach the clinic, it is believed that Piezo channel research will undoubtedly significantly influence GI disease treatment and will undoubtedly change clinical practice.
